# Proposed scenario for post-suicide attempt care for individuals undergoing gender transition

**DOI:** 10.1590/S2237-96222024v33e2024265.especial.en

**Published:** 2025-01-13

**Authors:** Vitória Alexandrina Volpe, Aline Conceição Silva, Laysa Fernanda Silva Pedrollo, Lucca de Oliveira Rosa, Manoel Antônio dos Santos, Elton Carlos Almeida, Kelly Graziani Giacchero Vedana

**Affiliations:** 1 Universidade de São Paulo, Departamento de Enfermagem Psiquiátrica e Ciências Humanas, Ribeirão Preto, SP, Brazil; 2 Universidade de São Paulo, Departamento de Enfermagem Materno-Infantil e Psiquiátrica, São Paulo, SP, Brazil; 3 Centro Universitário Municipal de Franca, Franca, SP, Brazil; 4 Faculdade de Filosofia, Ciências e Letras de Ribeirão Preto, Departamento de Psicologia, Ribeirão Preto, SP, Brazil; 5 Ministério da Saúde, Secretaria de Vigilância em Saúde e Ambiente, Brasília, DF, Brazil

**Keywords:** Personas Transgénero, Transexualidad, Prevención del Suicidio, Entrenamiento con Simulación de Alta Fidelidad, Atención en Salud Mental, Transgender People, Transsexuality, Suicide Prevention, High Fidelity Simulation Training, Mental Health Care

## Abstract

**Objective:**

To develop and validate a simulated scenario for post-suicide attempt care for a person undergoing gender transition.

**Method:**

This was a methodological study conducted in two stages: (i) development of the scenario based on the literature and clinical simulation recommendations; (ii) validation by ten experts, through an online assessment using a Likert scale and suggestions. Descriptive analyses and content validity index were applied.

**Results:**

**:** All items met the minimum acceptance criterion (≥0.70), and adjustments were made to incorporate a more inclusive approach and reflect the diversity of gender identities.

**Conclusion:**

The study developed and validated a clinical simulation scenario for post-suicide attempt care for a person undergoing gender transition. The expert validation indicated agreement on the scenario’s items, which can be applied in accessible training and qualification for healthcare professionals across different categories and contexts.

## Study contributions

**Main results:**

The scenario was developed based on scientific literature and validated by experts, with an emphasis on the complexities of gender transition and post-suicide attempt support. The scenario items met the minimum acceptance criteria.

**Implications for services:**

The validation of the simulated scenario supports professional training processes in health, specifically in mental health care and suicide prevention for the trans population, providing clear guidelines for practical application in health services.

**Perspectives:**

It is expected that training initiatives will be adopted for health professionals and students, using the scenario as a reference, in order to strengthen assistance and suicide prevention policies aimed at the trans population and promote research on appropriate care practices.

## INTRODUCTION

In 2019, it was estimated that approximately 703,000 people died by suicide, making it the fourth leading cause of death among young people aged 15 to 29 years that year.^1^ This issue is even more concerning in developing countries and among minority populations, such as the transgender community, which faces high levels of discrimination and violence, especially in contexts where institutional support and protection of human rights are limited.^2^

The lack of legal recognition and insufficient access to specialized healthcare, combined with prejudice and discrimination, expose this population to a high risk of social marginalization and compromised mental health, as reflected in increased suicide rates.^2^ Transgender people are those whose gender identity differs from that assigned at birth, including transsexual, transgender people and transvestites, which reflects a diversity of experiences that do not align with the social and biological expectations imposed by birth-assigned gender.^3^

A Brazilian study conducted with the trans population using social media highlighted that 70% of participants reported suicidal ideation, 40% attempted suicide and 55% presented depressive symptoms.^4^

In this context, the fragility of healthcare training to meet the demands of the trans population is highlighted. The literature underscores the inadequacies in health education regarding the care of transgender people and transvestites within the Brazilian health system. Studies highlight the neglect in the care of trans bodies, the lack of adequate support and the scarcity of discussions about their specific needs. In addition, they point to discrimination and unpreparedness of healthcare professionals, factors that contribute to the continued social and emotional vulnerability of this population.^5^ Looking at the Brazilian context, there are also significant gaps in professionals’ theoretical knowledge, particularly concerning the gender transition process and the basic rights of transgender people, which undermine the promotion of and access to healthcare for this group.^6^

Therefore, sharing strategies for suicide prevention within the transgender population must align with strengthening professional education, especially regarding the development of knowledge, attitudes, and skills. A safety plan or crisis management plan is a tool that can assist professionals in the care process, as it is a personalized and individual instrument that promotes self-knowledge, identification of distress, risk behaviors, protective factors, mental health promotion, and activation of support networks.^7,8^

In the contemporary scenario, clinical simulation is emerging as an innovative teaching and learning method aimed at training and qualifying professionals in various health settings. It is recognized for offering students and healthcare professionals a simulated experience of real-life care, establishing a solid link between theory and practice that promotes safe and responsible healthcare delivery.^9^ Through simulation, it becomes possible to identify and reflect on aspects in clinical practice that need improvement, based on experiences in simulated scenarios that can be developed at various levels of fidelity.^10^

This study adopted the term “gender transition” instead of “transsexualizing process” as it is considered more inclusive in describing the various experiences of gender identity. The term adopted encompasses a broad spectrum of experiences, including social, legal and/or medical changes, in contrast to “transsexualizing process”, which refers specifically to the medical treatment provided by the Brazilian National Health System (*Sistema Único de Saúde* - SUS). The objective of this study was to develop and validate a simulated scenario for post-suicide attempt care for a person undergoing gender transition. Thus, based on the context described, the guiding question of this study was outlined: “Is a simulated scenario for post-suicide attempt care for a person undergoing gender transition valid for teaching this topic?”

## METHODS

This was a methodological study conducted in two stages, (i) development, and (ii) validation of a simulated scenario. The development of the study was carried out based on the guidelines highlighted in the Methodological study reporting checklist.^11^

### Development of the simulated scenario

The scenario development began with the definition of the title, objectives and target population, ensuring that the simulation was appropriate to the participants’ level. Subsequently, the research team conducted a review of the scientific literature on crisis management plans,^8,12^ suicide prevention in the trans population,^13,14^ gender transition,^15^ as well as the National Policy for Comprehensive Health of Lesbians, Gays, Bisexuals, Transvestites and Transgender people (LGBT). Content published by the National Association of Transvestites and Transsexuals (ANTRA)^16^ was also analyzed, and the research team engaged with @transdiario channel.^17^ In addition, a technical visit was made to an outpatient clinic in an inland municipality in the state of São Paulo.

With this information, the research group developed a script for the scenario, aimed at assessing participants’ skills in clinical simulations, promoting reflective and structured learning. The preparation of the scenario included a pre -briefing to align expectations and reduce anxiety, in addition to organizing necessary resources, such as facilitators, environments and materials, and information for the simulated patient.

During the simulation, the objective structured clinical examination was used to analyze participants’ actions, which enabled a clear assessment and facilitated feedback on the performance of those involved. After the simulation, a structured was held conducted in three phases: the descriptive phase, which reviewed events without judgments; the analytical phase, which reflected on performance; and the application phase, which integrated the learning into clinical practice.^18,19^ This structure was based on international recommendations for designing high-fidelity simulated scenarios.^18^ After the scenario was developed, the research team conducted an internal evaluation to make minor adjustments and check spelling.

### Validation of the simulated scenario

The selection of experts to validate the scenario took place between March and October 2023. Using the Lattes platform’s search tool, experts were selected based on the study’s key topics. In separate searches for each topic, the keywords “transsexualizing process”, “transsexuality”, “suicidal behavior” and “high-fidelity simulation” were used. The search was conducted within the databases of PhDs and other researchers, with a focus on Brazilian nationality.

Despite using of filters, the results identified were broad, an aspect justified by the functionality and structure of the platform’s search presentation. Thus, the responsible researchers opted to select experts based on priority criteria and, from there, compile a list of possible contacts for research invitations. The defined criteria included having teaching experience in the area of interest, supervising academic work on the topic, holding a master’s or doctoral degree with research in the field, authoring scientific articles on the subject in journals classified by the Coordination for the Improvement of Higher Education Personnel (*Coordenação de Aperfeiçoamento de Pessoal de Nível Superior* - CAPES), and participating as an invited speaker at national or international scientific events related to the topic. In order to be selected the specialist had to meet at least one of these criteria defined for the study.

Based on this search, the curriculum data of 90 experts who met the proposed criteria were collected. The data were transcribed into an editable document, in list format, containing names and links to their curricula on the platform.

Initially, of the 90 experts selected, only 58 invitations were sent, of which eight experts agreed to participate in the study. Due to the small number of participants, the snowball sampling method was adopted, in which the initial participants indicate other potential participants, based on the same selection criteria. This resulted in an additional 29 invitations being sent, to which two new experts responded. Finally, validation was conducted with the participation of 10 experts with experience in clinical simulation, suicide prevention or gender transition processes.

The invitation to participate was sent via email, with a hyperlink to the online data collection platform, Research Electronic Data Capture (REDCap). A 21-day deadline was established for the experts to respond to the Free and Informed Consent Form and confirm their participation. A total of four experts responded that they could not participate in the research within that period; 73 were considered to have withdrawn, as they did not respond to the invitation or complete the research form.

For data collection in REDCap , two questionnaires were used, one to characterize the participants (age, sex, gender, region, academic background, years of professional experience and professional role) and the scenario script for evaluation, based on a three-point Likert scale (adequate, regular and inadequate) and spaces for suggestions. The scenario script consists of 13 topics, including title specification, general objectives, target population, human, physical, and material resources, prior study, duration, pre-briefing (information on contracts and simulation conduction), briefing (basic guidelines on the simulated case), instructions for the simulated patient, OSCE (expected and assessed items during the simulation), and debriefing (structured in three phases, according to The Diamond model).^20^

All data collected were organized, processed and analyzed in Microsoft Excel 10. To analyze the characterization data, simple descriptive statistics were performed; and, for the simulated scenario evaluation, the content validity index (CVI) was used, with an acceptance level of 70%.^21^

The project was approved by the Research Ethics Committee of the Escola de Enfermagem de Ribeirão Preto da Universidade de São Paulo, under opinion No. 5,204,375 and CAAE 53719221.7.0000.5393.

## RESULTS

The scenario was designed according to the learning objectives, addressing the following aspects: complexity of the gender transition process, weakening of the support network, difficulty in accessing specialized health services, seeking options that may pose health risks (such as using hormones without professional supervision), barriers to accessing rights (education, employment), experiences of discrimination and violence. Protective factors were also incorporated, including access to and follow-up by a qualified service and team, progress in self-understanding, positive outcomes in the gender transition process, and strengthened opportunities and support networks.

In addition to identifying these factors, the safety plan was used as a tool to guide the post-attempt support process (Supplementary Table).

The entire scenario was planned to reproduce aspects that are similar to the experiences of transgender people, aiming to encourage reflection and learning in the context of comprehensive health care.

A total of 10 experts participated in the study, the majority being cisgender men (n=5), with a mean age of 41.6 years (minimum=28, maximum=64, median=41.5 and standard deviation=9.7). Most experts lived in the Southeast (n=4), Midwest (n=4) and South (n=2) regions. Regarding the states, the participants were from São Paulo (n=3), the Federal District (n=3), Mato Grosso (n=1), Paraná (n=1), Rio de Janeiro (n=1) and Santa Catarina (n=1).

The predominant academic background among the experts was nursing (n=6), followed by psychology (n=2), social work (n=1) and medicine (n=1). The average length of professional experience was 12 years (minimum of 4, maximum of 40, median of 16 and standard deviation of 10.4). Most specialists worked as professors (n=6), in addition to participating in scientific research (n=5) and healthcare services (n=5). Regarding areas of expertise, most indicated knowledge in suicidal behavior (n=8), clinical simulation (n=5) and the gender transition process (n=5). As for acceptance and agreement, all items analyzed obtained CVI values equal to or greater than 0.72, reaching the minimum approval criterion (CVI=0.70), although some specialists did not respond to all items (Table 1).

**Table 1:** Acceptance and agreement of the validation by experts of a simulated scenario on post-suicide attempt support for a person undergoing gender transition, Brazil, 2023 (n=10)

Item	Agreement			Content Validity Index			
	**Adequate n**	**Fair**	**Inappropriate n**	**High fidelity simulation**	**Suicidal behavior**	**Transsexualizing process**	**Overall**
Title	5	5	-	1.00	1.00	1.00	1.00
Objective	5	5	-	1.00	1.00	1.00	1.00
Specific objectives	7	2		1.00	0.87	1.00	0.95
Target population	8	2	-	0.80	1.00	1.00	0.93
Number of People	10	-	-	1.00	1.00	1.00	1.00
Physical resources	8	1	1	0.80	1.00	1.00	0.93
Bibliography	8	1	-	0.80	0.87	0.80	0.82
Duration	6		-	1.00	1.00	1.00	1.00
Briefing	8	2	-	1.00	1.00	1.00	1.00
Instructions	8	2	-	1.00	1.00	1.00	1.00
Simulated Patient	8	-	1	0.80	0.75	0.60	0.72
OSCE^a^ 1	7	3	-	1.00	1.00	1.00	1.00
OSCE 2	8	1	1	0.80	1.00	1.00	0.93
OSCE 3	10	-	-	1.00	1.00	1.00	1.00
OSCE 4	9	1	-	1.00	1.00	1.00	1.00
OSCE 5	10	-	-	1.00	1.00	1.00	1.00
OSCE 6	10	-	-	1.00	1.00	1.00	1.00
OSCE 7	10	-	-	1.00	1.00	1.00	1.00
OSCE 8	9	-	1	0.80	1.00	1.00	0.93
Debriefing	9	1	-	1.00	1.00	1.00	1.00
References	10(100)	-	-	1.00	1.00	1.00	1.00

a) Objective Structured Clinical Examination.

Experts’ suggestions emphasized the importance of a careful and inclusive approach in the simulation process aimed at providing care for transgender men and transmasculine individuals. It was noted that not all trans people identify solely as “men,” and many prefer the term “transmasculine,” reflecting the diversity of gender experiences within this group. This recognition required adjustments to the simulation scenario to reflect the nuances of gender identity.

The experts also recommended including a transgender person or someone directly involved with the needs of this population as one of the observers of the simulation. One of them highlighted the importance of this participation, stating: “I suggest that one of the observers be a trans person or someone directly involved with this population and their main demands. I’m not sure of the feasibility of this suggestion, but I believe it’s important, especially to ensure the use of appropriate and non-sexist language, as inadequate language can be a trigger for distress and revictimization.”

Given that, adjustments were made to the instructions, in accordance with the experts’ suggestions, which caution against perpetuating binary gender norms rooted in society. One of the experts pointed out: “There are trans and cis men with long or short hair. Stating that the person representing the trans man should have short or tied-back hair is merely a reproduction of the binary logic, which I imagine this intervention aims to challenge.”

## DISCUSSION

The development of simulation in healthcare for the transgender population offers benefits related to improving the understanding of the challenges faced by this population.^22,23^ Thus, it is necessary to promote respect and inclusivity, ensure access to social rights (health, education, employment, and housing), and adopt a holistic view of health across all network services. it is crucial to avoid blaming the individual and instead focus on reflecting on care from the perspective of the need for structural changes in society, which marginalizes and excludes transgender people.

Simulated environments offer a safe space for the teaching-learning process, especially in the development of communication, empathy and support for the trans population.^22,23^

For the design of the scenario, specialized care services involved in the transsexualizing process under the SUS were chosen as the context. These services are part of the health care network and are responsible for outpatient actions, including clinical follow-up, pre- and post-operative care, and hormone therapy. The services are also supported by the National Policy for Comprehensive Health for Lesbians, Gays, Bisexuals, Transvestites, and Transgender people, which aims to promote the comprehensive health of the LGBT population by seeking to eliminate discrimination and institutional prejudice and contributing to reducing inequalities and consolidating SUS as a universal, integral, and equitable system.

Institutional attitudes can foster prejudice and discrimination, and weaken the healthcare relationship.^4,5^ Such situations are closely linked to gaps in professional training on gender identity issues, which are still superficially addressed in curricula and educational institutions.^24^

The scenario highlights risk factors in the lived experience of trans people, such as lack of support, indiscriminate use of hormones, violence^17^ and difficulty in accessing basic rights.^3^ Thus, it raises discussion of associated distress, not emphasizing the individual responsibility, but rather on social vulnerability as an important risk factor for health problems.

Protective factors were also incorporated, especially the strengthening of support networks^16^ and access to rights, such as housing, employment and health.^25^ A study conducted with transgender and non-binary youth revealed that follow-up and interventions in the transsexualizing process contributed to the reduction in depressive symptoms and suicidal thoughts during the first year of care.^26^ This finding is supported by other studies, which indicated that recognition of gender identity and identification of perceived needs during healthcare services fostered positive outcomes, increased self-care levels, and strengthened bonds with the healthcare environment.^4,5^

Given the limited discussion on the topics addressed in the simulated scenario, educational materials were provided for pre -briefing on the areas related to the transsexualizing process, suicidal behavior in the trans population^27^ and crisis management plan.^8^ The supplementary materials and references should be sent in advance for prior study by the participants in the clinical simulation activity, and can be supplemented by documents and policies that support this educational practice in the context where the scenario is applied.

In order to support the care process, the use of the crisis management plan or safety plan was selected, which presents positive characteristics for the continuity of care, since it provides professionals with relevant information for directing care, especially for risk analysis and implementation of strategies to manage crisis.^7,8^

Studies highlight the significant contributions of the safety plan in managing and preventing suicidal behavior.^7,8^ Prevention is directly linked to the management of emotions, anxiety, reduction of negative attitudes, and discrimination regarding the subject, in order to promote quality care that is tailored to the needs of people at risk of suicide.^28^

Recommendations for clinical simulation with simulated patients involve the description of appearance as an important component in constructing the patient. However, it is necessary to think about the construction of this information, in order to avoid reinforcing stigmatized and discriminatory structures. In this sense, the item on instructions for the simulated patient was restructured based on the experts’ suggestions, to avoid pathologizing of the transgender body and perpetuating the binary and transphobic logic.^7^ The construction of appearance was expanded in order to recognize sociocultural aspects. It is recommended that this topic be discussed during the debriefing to open dialogue on transmasculinity.

In the debriefing, the Diamond model was used, which allows recalling and clarifying clinical or technical issues, addressing perceptions and feelings, and reflecting on how to apply them in care or clinical practice.^20^ Thus, structured clinical objectives were prioritized to support the facilitator in guiding the debriefing, in alignment with the expected learning objectives in the simulated scenario.

The validation process aimed to evaluate and improve the scenario to better reflect the experiences of individuals, considering their historical-cultural context, needs, and the scientific evidence on the addressed topics. This reinforces the importance of reliable tools for teaching and clinical practice, such as the simulation script presented in this study. Simulation-based teaching can promote an education in which the student is positioned as the central axis of the training, and it is desirable that it be applied within a well-established pedagogical framework, with sufficient conditions for study, discussion and prior preparation, especially for the facilitators who will develop the scenario.^10^

A limitation of this study is the participation of mostly cisgender authors and experts, which restricted the scenario’s design based on the lived experiences of transgender people. In addition, there was a low number of experts involved and the absence of representatives from some regions of the country, such as the North and Northeast regions, which limits the diversity of perspectives. On the other hand, the study highlights the dialogue around the access of sexual and gender minorities to basic rights, such as the inclusion and permanence of trans people and other minority groups in educational and healthcare spaces.

In conclusion, this study developed and validated a clinical simulation scenario related to the post-suicide attempt care for a person undergoing gender transition. The validation performed by experts presented results that indicate agreement with the construction of the simulated scenario items. Thus, this article presents a fully validated simulation scenario, which can be used in an accessible and cost-effective manner for developing clinical simulation in the training of various professional categories, contributing to improving care in post-suicide attempt support for transgender individuals.

## Supplementary table

**Scenario Script for Post-Suicide Attempt Support for a Person Undergoing Gender Transition**
