# Supplementary material for: Proposed scenario for post-suicide attempt care for individuals undergoing gender transition
Source: Epidemiol Serv Saude. 2025 Jan 13;33(spe1):e2024265. doi: 10.1590/S2237-96222024v33e2024265.especial.en (PMC11734603; doi:10.1590/S2237-96222024v33e2024265.especial.en)
Supplement: Supplementary file 1 [file 2237-9622-ress-33-spe1-e2024265-supp01.pdf]

Supplementary table – Scenario Script for Post-Suicide Attempt Support for a Person Undergoing Gender Transition

Scenario title: Post-suicide attempt support for a person undergoing gender transition.

General objective *(objective to be achieved by participants in the simulated scenario).*
- To collaboratively develop a safety plan with a person undergoing gender transition.

Specific objectives *(objectives to be achieved by participants in the simulated scenario).*
- Address the initial needs presented by the trans man.
- Collaboratively build an individualized crisis management plan that addresses self-knowledge, well-being promotion, early crisis detection, help-seeking behavior, safety, and support

Target population for the scenario *(participants in the scenario).*
Undergraduate health students and health professionals (who have taken courses related to mental health/psychiatry).

Human resources *(Number of people needed to develop the scenario.)*
1. Two simulation facilitators (responsible for planning and coordinating the simulated activity).
2. Two participants (target population) who will provide assistance in the simulated scenario.
3. One simulated patient (will simulate the person treated in the scenario).
4. Observers (other participants).

Physical and material resources *(In this section, some basic items that could make up the scenario for carrying out the simulation are suggested).*
- A reception room of a health service (can be changed/adapted according to sociocultural context).
- Common objects that make up a reception room environment in a health service, such as: a table, two chairs, office supplies (pencils, pens, paper) and a lab coat.

Previous Study *(For simulation participants and observers; these materials will be provided by the simulation facilitators in advance via email for prior reading/viewing by all involved participants.)*

Transsexualizing Process
Trans Men & Transmasculinity Association (AHTM). Who Are Trans Men? [Online resource]. Available at: https://antrabrasil.files.wordpress.com/2018/01/cartilha-homens-trans-ahtm-versc3a3o-2-para-imprimir-e-distribuir-ao-pc3bablico-pdf.pdf.

Suicidal Behavior in the Trans Population
Benevides B. We Need to Talk About Suicide among Trans People! [Online resource]. 2018. Available at: https://antrabrasil.org/2018/06/29/precisamos-falar-sobre-o-suicidio-das-pessoas-trans/

Benevides B, et al. Suicide and Mental Health in the Trans Population. In: Dossier on Murders and Violence Against Brazilian Transvestites and Transgender Individuals in 2021. ANTRA; 2022. p. 98-101. Available at: https://antrabrasil.files.wordpress.com/2022/01/dossieantra2022-web.pdf

Crisis Management Plan
Silva AC, Vedana KGG. Crisis Management Plan. Online Tool [Internet]. Available at: https://inspiracao-leps.com.br/plano-de-gestao-de-crises/.

Silva AC, Mendonça A, Pedrollo LFS. Crisis Management Plan. Educational Video [Internet]. Available at: https://www.youtube.com/watch?v=sJ1EyFTNuOg

Wiche. Western Interstate Commission for Higher Education. Safety Planning Guide. 2008 [Internet]. Disponível em: https://www.sprc.org/sites/default/files/SafetyPlanningGuide%20Quick%20Guide%20for%20Clinicians.pdf

Duration (estimated time for each stage of the scenario):
Briefing (20 minutes).
Simulation (20 minutes).
Debriefing (40 minutes).

To be continued

Continuation

Supplementary table – Scenario Script for Post-Suicide Attempt Support for a Person Undergoing Gender Transition

**Pre-briefing** *(Information on contracts and conducting the simulation).*<br>Present the simulated environment to the scenario participants before the activity begins.<br>Discuss emotional safety with participants: confidentiality, anonymity, respect, and the importance of participation in the post-simulation discussion.<br>No handover or medical records will be presented or used for this simulated case.<br>Make it clear that, in the scenario, the activities will only take place there, with no interference from health professionals
**Briefing** *(presentation of the basic guidelines for the simulated case – these can be read and no information should be omitted).*<br><br>**This will be a simulation with a simulated patient**<br>Patient (social name): Luca - (trans man), 26 years old, single, mixed-race, self-employed, incomplete high school education, from São Paulo. Gender transition stage: undergoing hormone therapy for five months.<br>The patient reports that he was a victim of bullying in the past, which led him to drop out of high school. At 17, he was expelled from home by his parents, lived on the streets, and started using alcohol and tobacco. He self-administered hormones without medical supervision, leading to hypertensive crises, severe acne, joint pain, and tremors.<br>Five months ago, Luca sought care at the Specialized Care for the Transsexualizing Process. At first, he seemed quite nervous and had difficulty telling his story. He expressed his desire for specialized support for the transition process, as he had identified as a man since childhood. After evaluation, Luca began hormone therapy and follow-up by a multidisciplinary team at a specialized outpatient clinic.<br>He currently lives with friends, is employed by a recycled company, has started contact with non-governmental organizations (NGOs) that support trans people and has reestablished family ties only with his mother.<br>He reported that the gender transition process is very difficult, as the changes are slow and he still does not identify with his own body. He says he feels anxious because he has not yet undergone a total mastectomy, as this procedure is only authorized after two years of healthcare monitoring.<br>Five days ago, Luca attempted suicide by ingesting medication. He was treated in an Emergency Room and referred to a Psychosocial Care Center (*Centro de Atenção Psicossocial* - CAPS). He remains in outpatient care at the Gender Transition Clinic. This is Luca's first consultation after the suicide attempt at CAPS. You are the professionals responsible for Luca's care at CAPS and will help him develop a safety plan to manage crises. You have approximately 20 minutes to complete this activity. The simulation will not be interrupted by external individuals, and the facilitators will end the session when at least one healthcare team member completes the care or when the maximum execution time is reached. Do you have any questions about the instructions and preparation presented?

To be continued

Continuation

Supplementary table – Scenario Script for Post-Suicide Attempt Support for a Person Undergoing Gender Transition

Instructions for the simulated patient (*instructions for preparing the simulated patient for the case. Preparation should take place in the days leading up to the simulation).*

***Chief complaint*:** Patient does not identify with the gender assigned at birth (female). Five days ago, Luca attempted suicide by taking medication . (*refer to previous item: "Briefing - presentation of basic guidelines for the simulated case")*

Expected physical characteristics : Signs of distress or insomnia (example: dark circles under the eyes).

Main feelings and reactions:

Difficulty accepting his body and anxiety due to physical changes caused by hormone therapy
Example: "It's really bad, my body is changing very slowly, and I still have difficulty identifying with my body; I feel more anxious every day, I wish I could increase the hormone dosage and have a mastectomy."

Substance abuse
Example: "There are days when feelings get really intense; I feel so alone and I just want to drink, smoke cigarettes or marijuana to forget that I exist."

Relationship with support network
Examples: "I was taken in by friends, I have my job, NGOs help me share my pain, and currently my mother has been trying to reconnect with me. This gives me some comfort that I still have someone to rely on."

Suicide attempt
Refer to the following section: "Signs I experience when the most severe crisis approaches."

Signs for Crisis Management Plan construction
(The examples are flexible, that is, there may be several examples; these are just a few to guide the performer on the possibilities of choice when constructing the plan)

- **Signs that I am not doing well** – examples: I become pessimistic; anxious or distressed; emotional pain or sadness; tiredness or less energy; discouragement; guilt; anger at myself; dissatisfaction; I get irritated easily; frustration; lack of interest in things I like; lack of reasons to live; lack of purpose in life; feeling empty, reduced communication with others; changes in sleep patterns; changes in appetite; decreased performance in studies or work; increased use of alcohol and other legal or illegal drugs...

- **Examples of activities that help restore well-being** : Avoid making serious decisions; acknowledge small daily achievements; stay connected with people who bring positivity; recognize positive aspects of my life; talk to trusted individuals; maintain a healthy routine; engage in physical activities; relax; avoid self-criticism; enjoy leisure activities; meditate; connect with nature; read; listen to music; express feelings; reflect on things that bring joy; believe in the possibility of improvement with help; contact the *Centro de Valorização da Vida (CVV)* – 188; seek support from healthcare professionals or trusted individuals...

- **Signs that I experience when the most severe crisis approaches**: hopelessness; despair; intense or prolonged emotional pain; profound sadness; self-directed anger; impulsivity; frustration; feeling trapped; lack of motivation to live; emptiness; desire to self-harm; suicidal ideation or planning; engaging in risky behaviors...

- **Examples of actions to take a severe crisis approaches**: avoid making serious decisions; avoid being isolated; avoid places that could put me at risk; avoid things that I could use for self-harm; express my feelings; connect with support networks (SAMU – 192; FIRE DEPARTMENT – 193; POLICE – 190; telephone number of the nearest health service, health professional, etc.)

Note: The simulated patient must be familiar with the objective structured clinical examination (OSCE) (outlined below) before the role-play, so that he / she can incorporate his/her expected cues into the scenario.

To be continued

Continuation

Supplementary table – Scenario Script for Post-Suicide Attempt Support for a Person Undergoing Gender Transition

**OSCE** (*items to be considered in evaluating the performance of the scenario participants, according to the simulation objective(s).*<br>For each item below, assess whether the action was performed appropriately, using the response options YES, PARTIALLY or NO.	
**Items evaluated**	**Assessment**
Provide empathetic support without judgement.	( ) Yes<br>( ) partially<br>( ) no
Encourage autonomy and self-knowledge for crisis management, safety measures and risk reduction.	( ) Yes<br>( ) partially<br>( ) no
Help the person identify aspects that promote well-being.	( ) Yes<br>( ) partially<br>( ) no
Help the individual in recognizing early signs of a crisis.	( ) Yes<br>( ) partially<br>( ) no
Help the person identify healthy and safe coping strategies in crisis situations.	( ) Yes<br>( ) partially<br>( ) no
Help the person identify immediate specialized support resources (emergency services, specialized services, etc.).	( ) Yes<br>( ) partially<br>( ) no
Help the person identify the informal support network that can support them in difficult situations (friends, CVV, NGOs).	( ) Yes<br>( ) partially<br>( ) no
Schedule follow-up to reassess well-being, risk management and safety measures.	( ) Yes<br>( ) partially<br>( ) no
Debriefing *(Stage conducted post- scenario consisting of three consecutive phases, described below. All participants in the scenario – participants and observers – will be invited to reflect and discuss the simulation and experiences, knowledge, feelings and lessons learned in the simulated practice. Emphasis will be placed on items evaluated in the OSCE.*<br>***Debriefing based on "The Diamond" model***<br>*(JAYE, P.; THOMAS, L.; REEDY, G., 2015)*	
**Descriptive phase** *(focus on recounting what occurred in the case, without judging the participants'* performance during the simulation)<br>What happened during the initial welcoming of Luca? (Question directed to participants and observers of the scene).	
**Analytical phase** *(highlighting non-technical skills involved in the simulation that were crucial to the participants)*<br>How did you feel during the initial welcoming of Luca? Comment. (Question addressed to participants and observers in the scene).<br>How did you conduct the initial welcoming of Luca? (Question addressed to the observers).<br>How do you evaluate your group performance during the initial welcoming of Luca? (Question directed to participants in the scenario).<br>What positive actions were taken in the initial welcoming of Luca? (Question directed to participants and observers in the scenario).	
**Application phase** *(highlighting perspectives on how participants can apply knowledge in their clinical practice).*<br>What would you do differently during a future welcoming of Luca and crisis management after a suicide attempt? (Question directed to the participants in the scenario).<br>What lessons from this experience on welcoming Luca and addressing suicidal behavior in transgender individuals can you apply? (Question directed to participants and observers of the scenario).	

To be continued

Continuation

Supplementary table – Scenario Script for Post-Suicide Attempt Support for a Person Undergoing Gender Transition

References *(References used in the development of the scenario and which can be recommended as supplementary reading)*

Cabral CC, Muñoz NM. Uma transição compartilhada: sobre o acolhimento em saúde mental em um ambulatório do processo transexualizador do SUS. Rev Latinoam Psicopat Fund. 2021;24(2):259-280. https://doi.org/10.1590/1415-4714.2021v24n2p259.3

Dubov A, et al. Development of a smartphone app to predict and improve the rates of suicidal ideation among transgender persons (TransLife): qualitative study. J Med Internet Res. 2021;23(3). https://pubmed.ncbi.nlm.nih.gov/33596181/

Moscardini EH, et al. Suicide safety planning: clinician training, comfort, and safety plan utilization. Int J Environ Res Public Health. 2020;17(18):6444. https://doi.org/10.3390/ijerph17186444

Nuij C, van Ballegooijen W, et al. Safety planning-type interventions for suicide prevention: meta-analysis. Br J Psychiatry. 2021;219(2):419-426. https://doi.org/10.1192/bjp.2021.50

Oliveira ET, Vedana KGG. Suicídio e depressão na população LGBT: postagens publicadas em blogs pessoais. SMAD, Rev Eletrônica Saúde Mental Álcool Drog. 2020;16(4):39-48. http://dx.doi.org/10.11606/issn.1806-6976.smad.2020.168145

Pestana Gradim JG, Silva AC, Matias Pereira CC, Giacchero Vedana KG. Análisis de posturas sobre suicidio y comunidad LGBTQ en Twitter. Salud & Sociedad. 2020;10(3):286-294. https://revistas.ucn.cl/index.php/saludysociedad/article/view/3881

Popadiuk GS, Oliveira DC, Signorelli MC. A Política Nacional de Saúde Integral de Lésbicas, Gays, Bissexuais e Transgêneros (LGBT) e o acesso ao Processo Transexualizador no Sistema Único de Saúde (SUS): avanços e desafios. Cien Saude Colet [online]. 2017;22(5):1509-1520. https://doi.org/10.1590/1413-81232017225.32782016

Stanley B, Brown GK, Brenner LA, et al. Comparison of the safety planning intervention with follow-up vs usual care of suicidal patients treated in the emergency department. JAMA Psychiatry. 2018;75(9):894-900. https://doi.org/10.1001/jamapsychiatry.2018.1776
