# Supplementary material for: Proposed scenario for post-suicide attempt care for individuals undergoing gender transition
Source: Epidemiol Serv Saude. 2025 Jan 13;33(spe1):e2024265. doi: 10.1590/S2237-96222024v33e2024265.especial.en (PMC11734603; doi:10.1590/S2237-96222024v33e2024265.especial.en)
Supplement: Supplementary file 2 [file 2237-9622-ress-33-spe1-e2024265-supp01-pt.pdf]

Quadro suplementar – Roteiro do cenário simulado para acolhimento pós-tentativa suicida de uma pessoa em processo de transição de gênero

**Título do cenário:** Acolhimento pós-tentativa suicida de uma pessoa em processo de transição de gênero.
**Objetivo geral** *(objetivo a ser atingido pelos participantes do cenário simulado).*<br>- Realizar a construção conjunta de um plano de segurança com uma pessoa em processo de transição de gênero.
**Objetivos específicos** *(objetivos a serem atingidos pelos participantes do cenário simulado).*<br>- Acolher as necessidades iniciais apresentadas pelo homem trans.<br>- Construir, de forma colaborativa, um plano de gestão de crises individualizado, que aborde o autoconhecimento, promoção do bem-estar, detecção precoce de crises, busca de ajuda, segurança e suporte.
**Público-alvo do cenário** *(participantes do cenário).*<br>Alunos de graduação da área da saúde e profissionais da área da saúde (que tenham cursado disciplinas relacionadas à saúde mental/psiquiatria).
**Recursos humanos** *(Quantidade de pessoas necessárias para o desenvolvimento do cenário.)*<br>1. Dois facilitadores da simulação (responsáveis por planejar e coordenar a atividade simulada).<br>2. Dois participantes (público-alvo) que realizarão o atendimento no cenário simulado.<br>3. Um paciente simulado (simulará a pessoa atendida no cenário).<br>4. Observadores (demais participantes).
**Recursos físicos e materiais** *(Nesta seção, estão sugeridos alguns itens básicos que poderão compor o cenário para realização da simulação).*<br>- Uma sala de acolhimento de um serviço de saúde (pode ser alterada/adaptada conforme a realidade sociocultural).<br>- Objetos comuns que compõem um ambiente de sala de acolhimento de um serviço de saúde, tais como: uma mesa, duas cadeiras, materiais de escritório (lápis, canetas, papéis) e jaleco.
Estudo prévio *(Para os participantes e observadores da simulação; estes materiais serão disponibilizados pelos facilitadores da simulação, com antecedência e via* e-mail*, para a leitura/visualização prévia por todos os participantes envolvidos).*<br><br>**Processo transexualizador**<br>Associação de Homens Trans & Transmasculinidades (AHTM). Quem são os homens trans? Cartilha [Internet]. Disponível em: https://antrabrasil.files.wordpress.com/2018/01/cartilha-homens-trans-ahtm-versc3a3o-2-para-imprimir-e-distribuir-ao-pc3bablico-pdf.pdf<br><br>**Comportamento suicida na população trans**<br>Benevides B. Precisamos falar sobre o suicídio das pessoas trans! [Internet]. 2018. Disponível em: https://antrabrasil.org/2018/06/29/precisamos-falar-sobre-o-suicidio-das-pessoas-trans/<br><br>Benevides B, et al. Suicídio e a saúde mental da população trans. In: Dossiê Assassinatos e violências contra travestis e transexuais brasileiras em 2021. ANTRA; 2022. p. 98-101. Disponível em: https://antrabrasil.files.wordpress.com/2022/01/dossieantra2022-web.pdf<br><br>**Plano de Gestão de Crises**<br>Silva AC, Vedana KGG. Plano de Gestão de Crise. Ferramenta Online [Internet]. Disponível em: https://inspiracao-leps.com.br/plano-de-gestao-de-crises/<br><br>Silva AC, Mendonça A, Pedrollo LFS. Plano de Gestão de Crise. Vídeo Educativo [Internet]. Disponível em: https://www.youtube.com/watch?v=sJ1EyFTNuOg<br><br>Wiche. Western Interstate Commission for Higher Education. Safety Planning Guide. 2008 [Internet]. Disponível em: https://www.sprc.org/sites/default/files/SafetyPlanningGuide%20Quick%20Guide%20for%20Clinicians.pdf
**Tempo de duração** *(tempo estimado de duração de cada uma das etapas do cenário).*<br>*1.* *Briefing* (20 minutos).<br>2. Simulação (20 minutos).<br>*3.* *Debriefing* (40 minutos).

Continua

Continuação

Quadro suplementar – Roteiro do cenário simulado para acolhimento pós-tentativa suicida de uma pessoa em processo de transição de gênero

Pré-briefing *(Informações sobre contratos e condução da simulação).*<br>Realizar a apresentação do ambiente simulado para os participantes do cenário antes do início da atividade.<br>Discutir contratos sobre a segurança emocional com os participantes: sigilo, anonimato, respeito e a importância da participação na discussão posterior à simulação.<br>Para este caso simulado, não está prevista a passagem de plantão, leitura de prontuário da(o) usuária(o) e apresentação e/ou uso de prescrição medicamentosa.<br>Deixar claro que, no cenário, as atividades vão ocorrer somente ali, não havendo interferência de profissionais da saúde
Briefing *(apresentação das orientações básicas do caso simulado – poderão ser lidas e nenhuma das informações deve ser omitida).*<br><br>**Essa será uma simulação com um paciente simulado**<br>Paciente (nome social): Luca - (homem trans), 26 anos, solteiro, pardo, autônomo, ensino médio incompleto, natural de São Paulo. Etapa processo de transição: hormonioterapia há cinco meses.<br>Paciente relata que, no passado, foi vítima de *bullying*, o que culminou na evasão escolar no Ensino Médio. Aos 17 anos, foi expulso de casa pelos pais, tendo permanecido em situação de rua e iniciado o uso de álcool e tabaco. Fez uso de hormônios sem prescrição médica, o que o levou a ter picos de hipertensão, acne severa, dores nas articulações e tremores.<br>Há cinco meses, Luca procurou o atendimento na Atenção Especializada no Processo Transexualizador. No primeiro momento, se mostrou bastante nervoso e com dificuldade de contar sua história. Verbalizou o desejo de acompanhamento especializado para processo de transição, pois se identificava como homem desde sua infância. Após avaliação, Luca iniciou a hormonioterapia e o acompanhamento por equipe multidisciplinar em um ambulatório especializado.<br>Atualmente mora com amigos, tem vínculo empregatício em uma empresa de materiais reciclados, iniciou contato com organizações não governamentais (ONGs) de acolhimento a pessoas trans e reatou o vínculo familiar somente com a mãe.<br>Relatou que o processo de transição de gênero é muito difícil, pois as alterações são lentas e ele ainda não se identifica com o próprio corpo. Refere sentir-se ansioso por ainda não ter realizado a mastectomia total, pois este procedimento só é autorizado após dois anos de acompanhamento no sistema de saúde.<br>Há cinco dias, Luca fez uma tentativa suicida por ingestão medicamentosa, sendo acolhido no serviço de Urgência e Emergência e referenciado ao Centro de Atenção Psicossocial (CAPS). Permanece em acompanhamento ambulatorial na Atenção Especializada no Processo Transexualizador. Esta será a primeira consulta após a primeira tentativa suicida no CAPS.<br>Vocês são os profissionais responsáveis pelo acolhimento no CAPS e terão como objetivo acolher o Luca em suas necessidades, de modo a ajudá-lo a construir um plano de segurança para auxiliá-lo em seus momentos de crise. Vocês têm, aproximadamente, 20 minutos para realizar essa atividade.<br>A atividade de simulação não sofrerá a intervenção de pessoas externas ao cenário, e será finalizada pelos facilitadores da simulação quando ao menos uma pessoa da equipe de saúde finalizar o atendimento ou ao final do tempo máximo de execução. Vocês têm alguma dúvida sobre as orientações e preparo apresentados?

Continua

Continuação

Quadro suplementar – Roteiro do cenário simulado para acolhimento pós-tentativa suicida de uma pessoa em processo de transição de gênero

Instruções para o paciente simulado *(instruções que compõem o preparo do paciente simulado para atuar no caso simulado. O preparo deve ser realizado nos dias que antecedem a simulação).*

Queixa principal: Paciente não se identifica com o gênero atribuído ao nascimento (feminino). Há cinco dias, Luca fez uma tentativa suicida por ingestão medicamentosa. (*ver item anterior: "Briefing- apresentação das orientações básicas do caso simulado")*

Características físicas esperadas: Pode-se utilizar *facies* de sofrimento ou insônia (exemplo: olheiras).

Principais sentimentos e reações:

Dificuldade de aceitação do próprio corpo e ansiedade por modificações físicas da hormonioterapia
Exemplo: "É muito ruim, meu corpo está mudando muito devagar, e ainda tenho dificuldades de me identificar com meu corpo; me sinto cada dia mais ansioso, queria poder aumentar a quantidade de hormônios e realizar a mastectomia."

Uso abusivo de substâncias
Exemplo: "Tem dias que os sentimentos aparecem muito fortes; me sinto muito sozinho e só tenho vontade de beber, fumar um tabaco ou maconha para esquecer que eu existo."

Relação com rede de apoio
Exemplos: "Fui acolhido por amigos, tenho meu emprego, as ONGs me ajudam a compartilhar a minha dor, e atualmente minha mãe tem tentado se aproximar de mim. Isso me traz um certo conforto de que ainda tenho em quem me apoiar."

Tentativa suicida
Ver item a seguir: "Sinais que sinto quando a crise mais grave se aproxima."

Sinais para construção do Plano de Gestão de Crise
(Os exemplos são flexíveis, ou seja, pode haver diversos exemplos; esses são somente alguns para orientação do encenador sobre as possibilidades de escolha no momento da construção do plano)

- **Sinais de que não estou bem** – exemplos: fico pessimista; ansiedade ou angústia; dor emocional ou tristeza; cansaço ou com menos energia; desânimo; culpa; raiva de mim mesmo; insatisfação; fico irritado facilmente; frustração; desinteresse por coisa de que gosto; falta de motivos para viver; falta de sentido na vida; sensação de vazio, converso menos com as pessoas; alteração no sono; alteração no apetite; menor rendimento em estudo ou trabalho; aumento o uso de álcool e demais drogas lícitas ou ilícitas...

- **Exemplos do que ajuda a recuperar o bem-estar:** evitar tomar decisões sérias; reconhecer cada pequena conquista do meu dia a dia; não desistir de mim; evitar ficar isolado e manter contato com pessoas que me fazem bem; reconhecer aspectos positivos que tenho em minha vida; conversar com pessoas nas quais confio; tentar manter uma rotina mais saudável; descansar; realizar atividades físicas; não me cobrar demais; atividades de lazer, relaxamento; meditação; contato com a natureza; ler; ouvir música; expressar meus sentimentos; lembrar das coisas que me fazem bem; acreditar que com ajuda posso melhorar; entrar em contato com o Centro de Valorização da Vida (CVV) – 188; buscar ajuda das pessoas que me apoiam; buscar apoio de profissionais ou serviços de saúde...

- **Sinais que sinto quando a crise mais grave se aproxima:** desesperança; desespero; dor emocional forte ou duradoura; tristeza profunda; raiva de mim mesmo; impulsividade; frustração; me sinto sem saída; falta de motivos para viver; falta de sentido na vida; desejo de me machucar; desejo ou plano de me matar; comportamentos que me colocam em risco...

- **Exemplos do que fazer caso a crise mais grave se aproximar:** evitar tomar decisões sérias; evitar ficar isolado; evitar locais que possam me colocar em risco; evitar coisas que eu possa utilizar para fazer mal a mim mesmo; expressar meus sentimentos; conectar com redes de apoio (SAMU – 192; BOMBEIROS – 193; POLÍCIA – 190; telefone do serviço de saúde mais próximo, do profissional da saúde etc.)

Observação: é necessário que o paciente simulado conheça o exame clínico objetivo estruturado (ECOE) (item a seguir) antes da encenação, para que possa programar suas pistas de acordo com o que se espera do cenário.

Continua

Continuação

Quadro suplementar – Roteiro do cenário simulado para acolhimento pós-tentativa suicida de uma pessoa em processo de transição de gênero

ECOE (*itens a serem considerados na avaliação do desempenho dos participantes do cenário, conforme o(s) objetivo(s) da simulação*).
Para cada item a seguir, avalie se a ação realizada foi executada adequadamente, utilizando as opções de resposta SIM, PARCIALMENTE ou NÃO.

Itens avaliados	Avaliação
Realizar acolhimento de forma empática, sem julgamentos.	( ) sim<br>( ) parcialmente<br>( ) não
Incentivar a autonomia e o autoconhecimento para gestão de crises, medidas de segurança e redução de riscos.	( ) sim<br>( ) parcialmente<br>( ) não
Ajudar a pessoa a identificar aspectos que promovem o bem-estar.	( ) sim<br>( ) parcialmente<br>( ) não
Ajudar a pessoa a identificar sinais que precedem uma crise.	( ) sim<br>( ) parcialmente<br>( ) não
Ajudar a pessoa a identificar estratégias de enfrentamento saudáveis e seguras em situações de crise.	( ) sim<br>( ) parcialmente<br>( ) não
Ajudar a pessoa a identificar recursos de apoio especializado imediatos (serviço urgência e emergência, serviços especializados, etc.).	( ) sim<br>( ) parcialmente<br>( ) não
Ajudar a pessoa a identificar a rede de apoio informal que pode apoiá-la em situações difíceis (amigos, CVV, ONGs).	( ) sim<br>( ) parcialmente<br>( ) não
Agendar a reavaliação das medidas de bem-estar, gestão de risco e segurança.	( ) sim<br>( ) parcialmente<br>( ) não

Debriefing *(Etapa desenvolvida após o cenário por meio de três fases consecutivas, descritas a seguir. Neste momento, todos os participantes do cenário – participantes e observadores – serão convidados a refletir e dialogar sobre a simulação e experiências, conhecimentos, sentimentos e aprendizados envolvidos na prática simulada, com destaque para aspectos listados e avaliados nos itens do ECOE.*
Debriefing baseado no modelo "The Diamond"
(JAYE, P.; THOMAS, L.; REEDY, G., 2015)

Fase descritiva *(evidenciar olhares sobre o que ocorreu no caso, sem julgamentos sobre a* performance *dos participantes durante a simulação)*
O que aconteceu durante a realização do acolhimento inicial ao Luca? (Questão direcionada aos participantes e observadores do cenário).

Fase analítica *(evidenciar olhares sobre habilidades não técnicas envolvidas na simulação que foram importantes para os participantes)*
Como se sentiram durante a realização do acolhimento inicial ao Luca? Comentem. (Questão direcionada aos participantes e observadores do cenário).
Como realizaram o acolhimento inicial ao Luca? (Questão direcionada aos observadores).
Como consideram o seu desempenho no trabalho em grupo durante o acolhimento inicial ao Luca? (Questão direcionada aos participantes do cenário).
Quais ações positivas foram realizadas no acolhimento inicial ao Luca? (Questão direcionada aos participantes e observadores do cenário).

Fase aplicativa *(evidenciar olhares sobre como os participantes poderão aplicar o conhecimento em sua prática clínica).*
O que fariam diferente durante uma nova vivência de acolhimento ao Luca e acompanhamento em relação à gestão de crises e a sua segurança após tentativa suicida? (Questão direcionada aos participantes do cenário).
O que poderão levar dessa experiência sobre acolhimento ao Luca e o comportamento suicida na população transexual? (Questão direcionada aos participantes e observadores do cenário).

Continua

Continuação

Quadro suplementar – Roteiro do cenário simulado para acolhimento pós-tentativa suicida de uma pessoa em processo de transição de gênero

Referências *(Referências utilizadas na elaboração do cenário e que podem ser recomendadas como leitura complementar)*

Cabral CC, Muñoz NM. Uma transição compartilhada: sobre o acolhimento em saúde mental em um ambulatório do processo transexualizador do SUS. Rev Latinoam Psicopat Fund. 2021;24(2):259-280. https://doi.org/10.1590/1415-4714.2021v24n2p259.3

Dubov A, et al. Development of a smartphone app to predict and improve the rates of suicidal ideation among transgender persons (TransLife): qualitative study. J Med Internet Res. 2021;23(3). https://pubmed.ncbi.nlm.nih.gov/33596181/

Moscardini EH, et al. Suicide safety planning: clinician training, comfort, and safety plan utilization. Int J Environ Res Public Health. 2020;17(18):6444. https://doi.org/10.3390/ijerph17186444

Nuij C, van Ballegooijen W, et al. Safety planning-type interventions for suicide prevention: meta-analysis. Br J Psychiatry. 2021;219(2):419-426. https://doi.org/10.1192/bjp.2021.50

Oliveira ET, Vedana KGG. Suicídio e depressão na população LGBT: postagens publicadas em blogs pessoais. SMAD, Rev Eletrônica Saúde Mental Álcool Drog. 2020;16(4):39-48. http://dx.doi.org/10.11606/issn.1806-6976.smad.2020.168145

Pestana Gradim JG, Silva AC, Matias Pereira CC, Giacchero Vedana KG. Análisis de posturas sobre suicidio y comunidad LGBTQ en Twitter. Salud & Sociedad. 2020;10(3):286-294. https://revistas.ucn.cl/index.php/saludysociedad/article/view/3881

Popadiuk GS, Oliveira DC, Signorelli MC. A Política Nacional de Saúde Integral de Lésbicas, Gays, Bissexuais e Transgêneros (LGBT) e o acesso ao Processo Transexualizador no Sistema Único de Saúde (SUS): avanços e desafios. Cien Saude Colet [online]. 2017;22(5):1509-1520. https://doi.org/10.1590/1413-81232017225.32782016

Stanley B, Brown GK, Brenner LA, et al. Comparison of the safety planning intervention with follow-up vs usual care of suicidal patients treated in the emergency department. JAMA Psychiatry. 2018;75(9):894-900. https://doi.org/10.1001/jamapsychiatry.2018.1776
