# Supplementary material for: Proposed scenario for post-suicide attempt care for individuals undergoing gender transition
Source: Epidemiol Serv Saude. 2025 Jan 13;33(spe1):e2024265. doi: 10.1590/S2237-96222024v33e2024265.especial.en (PMC11734603; doi:10.1590/S2237-96222024v33e2024265.especial.en)
Supplement: Translation

# Translation: Proposta de cenário para acolhimento pós-tentativa de suicídio de pessoas em processo de transição de gênero

**DOI:** 10.1590/S2237-96222024v33e2024265.especial.pt

**Authors:** Vitória Alexandrina Volpe, Aline Conceição Silva, Laysa Fernanda Silva Pedrollo, Lucca de Oliveira Rosa, Manoel Antônio dos Santos, Elton Carlos Almeida, Kelly Graziani Giacchero Vedana

**Affiliations:** 1 Universidade de São Paulo, Departamento de Enfermagem Psiquiátrica e Ciências Humanas, Ribeirão Preto, SP, Brasil; 2 Universidade de São Paulo, Departamento de Enfermagem Materno-Infantil e Psiquiátrica, São Paulo, SP, Brasil; 3 Centro Universitário Municipal de Franca, Franca, SP, Brasil; 4 Faculdade de Filosofia, Ciências e Letras de Ribeirão Preto, Departamento de Psicologia, Ribeirão Preto, SP, Brasil; 5 Ministério da Saúde, Secretaria de Vigilância em Saúde e Ambiente, Brasília, DF, Brasil

**Keywords:** Pessoas Transgênero, Transexualidade, Prevenção de Suicídio, Treinamento com Simulação de Alta Fidelidade, Assistência à Saúde Mental

## Abstract

**Objetivo:**

Elaborar e validar um cenário simulado para o acolhimento pós-tentativa suicida de uma pessoa em transição de gênero.

**Método:**

Estudo metodológico realizado em duas etapas: (i) elaboração do cenário com base na literatura e recomendações de simulação clínica; (ii) validação por dez especialistas, utilizando avaliação *on-line* com escala Likert e sugestões. Análises descritivas e índice de validade de conteúdo foram aplicados.

**Resultados:**

Todos os itens atenderam ao critério mínimo de aceitação (≥0,70), e ajustes foram feitos para incluir uma abordagem mais inclusiva e refletir a diversidade de identidades de gênero.

**Conclusão:**

O estudo elaborou e validou um cenário de simulação clínica para o acolhimento pós-tentativa de suicídio de uma pessoa em processo de transição de gênero. A validação conduzida por especialistas indicou concordância quanto aos itens do cenário, que pode ser aplicado de forma acessível na formação e qualificação em saúde de profissionais de diferentes categorias e contextos.

## Contribuições do estudo

**Principais resultados:**

O cenário foi elaborado com base na literatura científica e validado por especialistas, com ênfase para as complexidades da transição de gênero e acolhimento pós-tentativa de suicídio. Os itens do cenário atenderam ao critério mínimo de aceitação.

**Implicações para os serviços:**

A validação do cenário simulado favorece os processos de formação profissional em saúde, especificamente no cuidado em saúde mental e na prevenção de suicídios na população trans, oferecendo diretrizes claras para a aplicação prática nos serviços de saúde.

**Perspectivas:**

Espera-se que sejam adotadas iniciativas de capacitação para profissionais e estudantes da saúde, utilizando o cenário como referência, a fim de fortalecer políticas de assistência e prevenção ao suicídio na população trans e fomentar pesquisas sobre cuidados adequados.

## INTRODUÇÃO

Em 2019, foi estimado que aproximadamente 703 mil pessoas morreram por suicídio, sendo esta a quarta principal causa de morte entre jovens de 15 a 29 anos naquele ano.^1^ Essa questão se torna mais preocupante em países em desenvolvimento e entre populações minoritárias, como a comunidade transexual, que enfrenta altos níveis de discriminação e violência, especialmente em contextos em que o apoio institucional e a proteção aos direitos humanos são limitados.^2^

A ausência de reconhecimento legal e o acesso insuficiente a cuidados de saúde especializados, associados ao preconceito e à discriminação, expõem essa população a um risco elevado de marginalização social e comprometimento da saúde mental, refletido no aumento das taxas de suicídio.^2^ Pessoas transexuais são aquelas cuja identidade de gênero difere da atribuída no nascimento, abrangendo indivíduos transexuais, transgêneros e travestis, o que reflete uma diversidade de vivências que não se enquadram nas expectativas sociais e biológicas impostas pela designação de gênero ao nascer.^3^

Um estudo brasileiro realizado com a população trans que utiliza a rede social destacou que 70% dos participantes mencionaram ideação suicida, 40% tentaram suicídio e 55% apresentaram sintomas depressivos.^4^

Nessa perspectiva, ressalta-se a fragilidade do processo formativo em saúde para atender às demandas da população trans. A literatura evidencia a fragilidade do processo formativo em saúde no que tange ao atendimento de pessoas transexuais e travestis no sistema de saúde brasileiro. Estudos destacam a negligência no cuidado a corpos trans, a falta de acolhimento adequado e a escassez de discussões sobre suas especificidades. Além disso, apontam para a discriminação e o despreparo dos profissionais de saúde, fatores que contribuem para a manutenção da vulnerabilidade social e emocional dessa população.^5^ Ao olharmos para o contexto brasileiro, é possível perceber também lacunas significativas no conhecimento teórico dos profissionais, especialmente em relação ao processo de transição de gênero e aos direitos básicos dos usuários, o que compromete a promoção e o acesso à saúde para esse segmento.^6^

Sendo assim, o compartilhamento de estratégias para a prevenção do comportamento suicida na população trans caminha em conjunto com a necessidade de fortalecimento da formação profissional, principalmente no que se refere à construção de conhecimentos, atitudes e habilidades. O plano de segurança ou plano de gestão de crises é uma ferramenta que pode auxiliar o profissional no processo de acolhimento, pois é um instrumento personalizado e individual que promove o autoconhecimento, identificação de mal-estar, comportamentos de risco, fatores protetivos, promoção da saúde mental e acionamento de redes de apoio.^7,8^

No cenário contemporâneo, a simulação clínica desponta como um método inovador de ensino e aprendizagem voltado para a formação e a capacitação de profissionais em diversos contextos de saúde. Reconhecida por possibilitar ao estudante e ao profissional de saúde uma experiência simulada da realidade da assistência, a simulação clínica estabelece uma relação sólida entre teoria e prática, que estimula a oferta do cuidado em saúde de forma segura e responsável.^9^ Por meio da simulação, torna-se possível a identificação e reflexão de pontos a serem aprimorados na prática clínica, a partir de vivências em cenários simulados que podem ser desenvolvidos em variados níveis de fidelidade.^10^

Este estudo adotou o termo “transição de gênero” ao invés de “processo transexualizador”, por considerar seu uso mais inclusivo na descrição das diversas experiências de identidade de gênero. O termo adotado abrange um espectro amplo de vivências, incluindo mudanças sociais, legais e/ou médicas, ao contrário do “processo transexualizador”, que se refere especificamente ao tratamento médico oferecido pelo Sistema Único de Saúde (SUS) no Brasil. O objetivo do estudo foi elaborar e validar um cenário simulado para o acolhimento pós-tentativa suicida de uma pessoa em transição de gênero. Dessa forma, a partir do contexto descrito, foi delineada a questão norteadora do estudo: “Um cenário simulado para o acolhimento pós-tentativa suicida de uma pessoa no processo de transição de gênero é válido para o ensino dessa temática?”.

## MÉTODOS

Trata-se de uma pesquisa metodológica realizada em duas etapas, (i) elaboração, e (ii) validação de um cenário simulado. O desenvolvimento do estudo foi realizado a partir das orientações destacadas no *Methodological study reporting checklist*.^11^

### Elaboração do cenário simulado

O desenvolvimento do cenário começou com a definição do título, dos objetivos e do público-alvo, assegurando que a simulação fosse adequada ao nível dos participantes. Em seguida, a equipe de pesquisa realizou um levantamento da literatura científica sobre plano de gestão de crises,^8,12^ prevenção do suicídio na população trans,^13,14^ transição de gênero,^15^ bem como da Política Nacional de Saúde Integral de Lésbicas, Gays, Bissexuais, Travestis e Transexuais (LGBT). Também foram analisados conteúdos publicados pela Associação Nacional de Travestis e Transexuais (Antra)^16^ e a equipe se aproximou do canal @transdiario.^17^ Além disso, foi realizada uma visita técnica a um ambulatório em um município do interior do estado de São Paulo.

Com essas informações, o grupo de pesquisa elaborou um roteiro para o cenário, que tinha como objetivo captar e avaliar as competências dos participantes em simulações clínicas, promovendo um aprendizado reflexivo e estruturado. A preparação do cenário incluiu um *pré-briefing* para alinhar expectativas e reduzir a ansiedade, além da organização dos recursos necessários, como facilitadores, ambientes e materiais, e informações para o paciente simulado.

Durante a simulação, foi utilizado o exame clínico objetivo estruturado como base para analisar as ações dos participantes, o que possibilitou uma avaliação clara e facilitou o *feedback* sobre o desempenho dos envolvidos. Após a simulação, realizou-se um *debriefing* estruturado em três fases: a fase descritiva, que revisou os eventos sem julgamentos; a fase analisadora, que refletiu sobre o desempenho; e a fase aplicativa, que integrou o aprendizado à prática clínica.^18,19^ Essa estrutura foi fundamentada em recomendações internacionais sobre o desenho de cenários simulados de alta fidelidade.^18^ Após a elaboração do cenário, o grupo de pesquisa conduziu uma avaliação interna para realizar pequenos ajustes e correções ortográficas.

### Validação do cenário simulado

A seleção de especialistas para a validação do cenário aconteceu entre os meses de março e outubro de 2023. A partir da ferramenta de busca na plataforma Lattes, foram selecionados especialistas nas temáticas focalizadas no estudo. No item assunto, em buscas separadas para cada tema, foram utilizadas as palavras-chave “processo transexualizador”, “transexualidade”, “comportamento suicida” e “simulação de alta fidelidade” A busca foi realizada nas bases de doutores e demais pesquisadores, com opção de nacionalidade brasileira. 

Apesar do uso de filtros, os resultados identificados foram amplos, aspecto justificado pela funcionalidade e estruturação na apresentação de resultados de busca na plataforma. Dessa forma, os pesquisadores responsáveis optaram por selecionar especialistas de acordo com critérios prioritários, e, a partir disso, compor uma lista de contatos possíveis para encaminhamento do convite de pesquisa. Os critérios definidos foram apresentar experiência docente na área de interesse, orientação de trabalhos acadêmicos sobre o tema, titulação de mestre ou doutor com trabalhos na área, autoria de artigos científicos sobre o tema em periódicos classificados pela Coordenação de Aperfeiçoamento de Pessoal de Nível Superior e participação como palestrante convidado em eventos científicos nacionais ou internacionais relacionados ao assunto. Para a sua seleção, o especialista deveria contemplar, no mínimo, um desses critérios definidos para o estudo. 

A partir da busca, foram coletados os dados dos currículos de 90 especialistas que estiveram em consonância com os critérios propostos. Os dados foram transcritos para um documento editável, no formato de lista, contendo nome e *link* de acesso ao currículo na plataforma. 

Inicialmente, dos 90 especialistas selecionados, apenas foram enviados 58 convites, dos quais oito especialistas aceitaram participar da pesquisa. Devido ao número reduzido de participantes, foi adotado o método de amostragem em bola de neve, no qual os participantes iniciais indicam outros possíveis participantes, baseado nos mesmos critérios de seleção. Isso resultou no envio de mais 29 convites, aos quais dois novos especialistas responderam. Ao final, a validação foi realizada com a participação de 10 especialistas com experiência em simulação clínica, prevenção do suicídio ou processo de transição de gênero. 

O convite para participação foi realizado via *e-mail*, com encaminhamento do *hiperlink* para a plataforma *online* de coleta de dados, o Research Electronic Data Capture (REDCap). Foi estabelecido o prazo de 21 dias para o retorno dos especialistas para o aceite do Termo de Consentimento Livre e Esclarecido e sua participação. No total, quatro especialistas responderam que não poderiam participar da pesquisa naquele período; 73 foram considerados desistentes, pois não retornaram o convite de participação ou não concluíram o formulário de pesquisa.

Para a coleta no REDCap, foram utilizados dois questionários, sendo um sobre caracterização dos participantes (idade, sexo, gênero, região, formação acadêmica, tempo de experiência profissional e atuação profissional) e o próprio roteiro do cenário para avaliação, a partir de escala Likert de três pontos (adequado, regular e inadequado) e espaços para sugestões. O roteiro do cenário é composto por 13 tópicos, com especificação do título, objetivo geral, público-alvo, recursos humanos, físicos e materiais, estudo prévio, tempo de duração, *pré-briefing* (informações sobre contratos e condução da simulação), *briefing* (orientações básicas sobre o caso simulado), instruções para o(a) paciente simulado(a), ECOE (itens esperados e avaliados durante a simulação) e *debriefing* (estruturado em três fases. conforme o modelo The Diamond).^20^

Todos os dados obtidos foram organizados, tratados e analisados no Microsoft Excel 10. Para análise dos dados de caracterização, foi realizada estatística descritiva simples; e, para avaliação do cenário simulado, optou-se pela utilização do índice de validade de conteúdo (IVC), com nível de aceitação em 70%.^21^

O projeto foi aprovado pelo Comitê de Ética em Pesquisa da Escola de Enfermagem de Ribeirão Preto da Universidade de São Paulo, sob o parecer nº 5.204.375 e CAAE 53719221.7.0000.5393. 

## RESULTADOS

O cenário foi delineado conforme os objetivos de aprendizagem; sendo assim, apresentou os seguintes aspectos: complexidade do processo de transição de gênero, fragilização da rede de apoio, dificuldade de acesso a serviços de saúde especializados, busca por opções que podem apresentar risco à saúde (uso de hormônios sem acompanhamento profissional), dificuldade de acesso a direitos (escola, emprego), discriminação e violência. Também foram inseridos fatores de proteção, como o acesso e acompanhamento por serviço e equipe qualificada, conquistas no processo de compreensão de si (*self*), resultados positivos no processo de transição de gênero, fortalecimento de oportunidades e redes de apoio. 

Além da identificação desses fatores, o plano de segurança foi utilizado como uma ferramenta para nortear o processo de acolhimento pós-tentativa (Quadro suplementar). 

Toda a elaboração do cenário foi planejada para reproduzir aspectos que se aproximam da vivência de pessoas transexuais, visando suscitar a reflexão e aprendizagem do cuidado ampliado em saúde. 

Participaram do estudo 10 especialistas, sendo a maioria homens cisgênero (n=5), com média de idade de 41,6 anos (mínima=28, máxima=64, mediana=41,5 e desvio-padrão=9,7). A maior parte dos especialistas residia nas regiões Sudeste (n=4), Centro-Oeste (n=4) e Sul (n=2). Em relação aos estados, os participantes eram de São Paulo (n=3), do Distrito Federal (n=3), de Mato Grosso (n=1), do Paraná (n=1), do Rio de Janeiro (n=1) e de Santa Catarina (n=1).

A formação acadêmica predominante entre os especialistas foi enfermagem (n=6), seguida de psicologia (n=2), serviço social (n=1) e medicina (n=1). O tempo médio de atuação profissional foi de 12 anos (mínima de 4, máxima de 40, mediana de 16 e desvio-padrão de 10,4). A maioria dos especialistas atuava como docente (n=6), além de participarem da pesquisa científica (n=5) e da assistência em saúde (n=5). Em relação à especialização, a maioria indicou ter conhecimento na temática do comportamento suicida (n=8), na simulação clínica (n=5) e no processo de transição de gênero (n=5). No que tange à aceitação e concordância, todos os itens analisados obtiveram valores de IVC iguais ou superiores a 0,72, atingindo o critério mínimo de aprovação (IVC=0,70), ainda que alguns especialistas não tenham respondido a todos os itens (Tabela 1).

**Tabela 1:** Aceitação e concordância da validação por especialistas de um cenário simulado sobre acolhimento pós-tentativa suicida de uma pessoa em processo de transição de gênero, Brasil, 2023 (n=10)

Item	Concordância			Índice de Validade de Conteúdo			
	Adequado n	Regular n	Inadequado n	Simulação de alta fidelidade	Comportamento suicida	Processo transexualizador	Geral
Título	5	5	-	1,00	1,00	1,00	1,00
Objetivo	5	5	-	1,00	1,00	1,00	1,00
Objetivos específicos	7	2		1,00	0,87	1,00	0,95
Público-alvo	8	2	-	0,80	1,00	1,00	0,93
Quantidade Pessoas	10	-	-	1,00	1,00	1,00	1,00
Recursos físicos	8	1	1	0,80	1,00	1,00	0,93
Bibliografia	8	1	-	0,80	0,87	0,80	0,82
Duração	6		-	1,00	1,00	1,00	1,00
Briefing	8	2	-	1,00	1,00	1,00	1,00
Instruções	8	2	-	1,00	1,00	1,00	1,00
Paciente Simulado	8	-	1	0,80	0,75	0,60	0,72
ECOE^a^ 1	7	3	-	1,00	1,00	1,00	1,00
ECOE 2	8	1	1	0,80	1,00	1,00	0,93
ECOE 3	10	-	-	1,00	1,00	1,00	1,00
ECOE 4	9	1	-	1,00	1,00	1,00	1,00
ECOE 5	10	-	-	1,00	1,00	1,00	1,00
ECOE 6	10	-	-	1,00	1,00	1,00	1,00
ECOE 7	10	-	-	1,00	1,00	1,00	1,00
ECOE 8	9	-	1	0,80	1,00	1,00	0,93
*Debriefing*	9	1	-	1,00	1,00	1,00	1,00
Referências	10(100)	-	-	1,00	1,00	1,00	1,00

a) Exame Clínico Objetivo Estruturado.

As sugestões dos especialistas concentraram-se na importância de uma abordagem cuidadosa e inclusiva no processo de simulação destinado ao atendimento de homens trans e pessoas transmasculinas. Foi destacado que nem todas as pessoas trans se identificam exclusivamente como “homens”, e que muitos preferem a identificação com o termo “transmasculino”, refletindo a diversidade de experiências de gênero dentro desse grupo. Esse reconhecimento demandou ajuste no cenário de simulação para refletir as nuances de identidade de gênero.

Os especialistas também sugeriram a inclusão de uma pessoa trans ou alguém diretamente envolvido com as demandas desse público entre os observadores da simulação. Um deles destacou a importância dessa participação, afirmando: “Sugiro que um dos observadores seja uma pessoa trans ou alguém diretamente envolvido com esse público e suas principais demandas. Não sei da viabilidade dessa sugestão, mas penso ser importante, especialmente para garantir o uso de uma linguagem adequada e não sexista, já que o uso de uma linguagem inadequada pode ser um gatilho para sofrimento e causar revitimização”.

Diante dessas considerações, foram feitas adequações nas instruções, conforme as sugestões dos especialistas, que alertam para a reprodução da lógica binária enraizada na sociedade. Um dos especialistas pontuou: “Há homens trans e cis com cabelos longos ou curtos. Demarcar que o cabelo de quem representará o homem trans seja curto ou esteja preso é mera reprodução da lógica binária, que eu imagino estar sendo combatida por esta intervenção”.

## DISCUSSÃO 

O desenvolvimento da simulação no cuidado em saúde da população transexual traz benefícios em aspectos relacionados à melhora da compreensão dos problemas enfrentados por essa população.^22,23^ Dessa forma, é necessário trabalhar o respeito e acolhimento, o acesso aos direitos sociais (saúde, educação, emprego e habitação) e a visão holística em saúde, em todos os dispositivos da rede. E, especialmente, evitar a culpabilização do indivíduo, fazendo-se uma reflexão acerca do cuidado, a partir da necessidade de mudança na estrutura social, que marginaliza e exclui pessoas transexuais.

Ambientes simulados oferecem um espaço seguro para o processo de ensino-aprendizagem, especialmente no desenvolvimento da comunicação, empatia e acolhimento à população trans.^22,23^

Para elaboração do cenário, foi elencado como contexto os serviços de atenção especializada no processo transexualizador amparado pelo SUS. Tais serviços integram a rede de assistência em saúde e são responsáveis por ações de natureza ambulatorial, desde o acompanhamento clínico, pré e pós-operatório e hormonioterapia. Os serviços também são resguardados pela Política Nacional de Saúde Integral de Lésbicas, Gays, Bissexuais, Travestis e Transexuais, que visa promover a saúde integral da população LGBT, buscando eliminar a discriminação e o preconceito institucional e contribuindo para a redução das desigualdades e consolidação do SUS como sistema universal, integral e equitativo. 

Atitudes institucionais podem promover preconceito e discriminação, e enfraquecer o vínculo de cuidado em saúde.^4,5^ Tais situações estão intimamente imbricadas com a fragilização da formação profissional nas questões de identidade de gênero, ainda tratada de forma superficial nos currículos e instituições de ensino.^24^

O cenário destaca fatores de risco na vivência de pessoas trans, como a ausência de suporte, uso indiscriminado de hormônios, violência^17^ e dificuldade no acesso a direitos básicos.^3^ Assim, suscita a discussão sobre o sofrimento, não enfatizando a responsabilização do indivíduo, mas partindo da vulnerabilização social como importante fator de risco para os agravos em saúde. 

Também foram incorporados fatores de proteção, especialmente o fortalecimento da rede de apoio ^16^ e acesso a direitos, como moradia, emprego e saúde.^25^ Um estudo realizado com jovens transgêneros e não binários revelou que o acompanhamento e intervenções no processo transexualizador contribuíram para a redução de sintomas depressivos e pensamentos suicidas durante o primeiro ano de atendimento.^26^ Tal achado é reforçado por outras pesquisas, que apontaram que o reconhecimento da identidade de gênero e a identificação das necessidades percebidas durante a assistência em saúde favoreceram resultados positivos, incrementando os níveis de autocuidado e fortalecendo o vínculo com o ambiente de saúde.^4,5^

Frente à limitação de diálogo sobre as temáticas que envolvem o cenário simulado, foram elencados materiais educativos para o *pré-briefing* sobre as áreas do processo transexualizador, comportamento suicida na população trans^27^ e plano de gestão de crises.^8^ Os materiais e referências complementares devem ser enviados com antecedência mínima para estudo prévio dos participantes da atividade de simulação clínica, e podem ser complementados por documentos e políticas que apoiem essa prática educativa no contexto onde o cenário for aplicado.

Para colaborar no processo de acolhimento, foi selecionado o uso do plano de gestão de crises ou plano de segurança, que apresenta características positivas para a continuidade da assistência, visto que propicia aos profissionais informações relevantes para o direcionamento do cuidado, em especial, para análise de riscos e implementação de estratégias para o manejo dos momentos de crise.^7,8^

Estudos destacam importantes contribuições do uso do plano de segurança no manejo e prevenção do comportamento suicida.^7,8^ A prevenção está diretamente ligada com o manejo de emoções, ansiedade, redução de atitudes negativas, discriminação acerca do assunto, de modo a promover um cuidado de qualidade e singularizado às necessidades das pessoas em risco de suicídio.^28^

As recomendações sobre simulação clínica com pacientes simulados envolvem a descrição da aparência como um componente importante para a construção do paciente. Entretanto, é necessário pensar na construção dessas informações, de forma a evitar o reforço de estruturas estigmatizadas e discriminatórias. Nesse sentido, o item sobre as instruções ao paciente simulado foi reestruturado a partir das sugestões dos especialistas, para evitar a patologização do corpo trans e a consequente reprodução da lógica binária e transfóbica.^7^ A construção da aparência foi ampliada de forma a reconhecer aspectos socioculturais. Recomenda-se discutir tal aspecto no *debriefing*, de forma a abrir o diálogo sobre as transmasculinidades.

No *debriefing*, foi utilizado o modelo *The Diamond*, que permite relembrar e esclarecer questões clínicas ou técnicas, trabalhar percepções e sentimentos e refletir sobre a aplicação na assistência ou prática clínica.^20^ Assim, foram priorizados objetivos clínicos estruturados que dão suporte ao facilitador na condução do *debriefing,* em diálogo com os objetivos esperados para aprendizagem no cenário simulado. 

O processo de validação foi realizado com o intuito de avaliar e aprimorar o cenário, de modo que se assemelhe às experiências de pessoas, considerando seu contexto histórico-cultural, suas necessidades e as evidências científicas sobre as temáticas trabalhadas. Com isso, reforça-se a importância de ferramentas confiáveis para o ensino e a prática clínica, como o roteiro de simulação apresentado no presente estudo. O ensino baseado na simulação pode promover uma educação na qual o estudante é posicionado como o eixo central de sua formação, e é desejável que seja aplicado dentro de uma proposta pedagógica bem estabelecida, com condições suficientes de estudo, discussões e preparo prévio, principalmente dos facilitadores que desenvolverão o cenário.^10^

Destaca-se como limitação desta pesquisa a participação de autores e especialistas em sua maioria cisgêneros, o que restringiu a elaboração frente às experiências de vida de pessoas trans. Além disso, houve um número baixo de especialistas envolvidos e a ausência de representantes de algumas regiões do país, como Norte e Nordeste, o que limita a diversidade de perspectivas. Em contrapartida, o estudo reforça o diálogo em torno do acesso das minorias sexuais e de gênero a direitos básicos, como a inserção e permanência de pessoas trans e de outros grupos minoritários em espaços de ensino e assistência. 

Em conclusão, este estudo elaborou e validou um cenário de simulação clínica relacionado ao acolhimento pós-tentativa suicida de uma pessoa no processo de transição de gênero. A validação realizada por especialistas apresentou resultados que indicam concordância em relação à construção dos itens do cenário simulado. Assim, o artigo apresenta, na íntegra, um cenário simulado validado, que pode ser empregado de forma acessível e econômica para o desenvolvimento da simulação clínica na formação de diferentes categorias profissionais, de modo a colaborar na qualificação da assistência no acolhimento pós-tentativa suicida de uma pessoa transexual.

## Quadro suplementar

**Roteiro do cenário simulado para acolhimento pós-tentativa suicida de uma pessoa em processo de transição de gênero**
